# Characterization of different virulent factors in methicillin-resistant *Staphylococcus aureus* isolates recovered from Iraqis and Syrian refugees in Duhok city, Iraq

**DOI:** 10.1371/journal.pone.0237714

**Published:** 2020-08-17

**Authors:** Narin A. Rasheed, Nawfal R. Hussein

**Affiliations:** 1 Department of Medical Microbiology, College of Medicine, University of Duhok, Kurdistan Region, Iraq; 2 Akre Technical Institute, Duhok Polytechnic University, Duhok, Kurdistan Region, Iraq; 3 Department of Medicine, College of Medicine, University of Zakho, Zakho, Kurdistan Region, Iraq; Instituto de Technologia Quimica e Biologica, PORTUGAL

## Abstract

Methicillin-resistant *Staphylococcus aureus* (MRSA) is a serious public health problem. There is limited information regarding the genetics of MRSA strains among the native Iraqi and incoming Syrian refugee communities. We aimed to characterize the genotypes and different virulence factors of MRSA in strains isolated from these two communities. Frozen MRSA strains (125) isolated from the native Iraqi and Syrian refugee communities were used in this study. PCR (singleplex and multiplex) and *agr* typing was used for the genotypic analysis of different virulence genes. We tested for the presence of virulence genes including *pvl*, *arc*A, *tst*, *lukE/lukD*, *hla*, *hlb*, *eta*, *etb* and *agr*. Prevalence of *arc*A MRSA in the Iraqi community (56.58%) was significantly higher (p = 0.008) than that in the Syrian refugee community (32.66%). Prevalence of *lukE-lukD* was also significantly higher (*p* = 0.001) in the Iraqi (82.89%) compared to that in the Syrian refugee community (57.14%). Further, prevalence of *hla* MRSA in the Iraqi community was (93.4%) and in the Syrian refugee community was (71.4%); (*p* = 0.0008). No significant differences were observed in the prevalence of *pvl*, *tst*, *eta*, *etb* and *hlb*. The most dominant *agr* types in both Iraqi (76.1% and 10.5%) and Syrian refugee (44.9% and 18.37%) communities were I and III. To sum up, no significant differences were observed between the groups for a majority of virulence factors. This is the first investigation of MRSA genotypes and virulence in both these communities. These results could be useful for further studies that assess the genetic relatedness of strains in the region for epidemiological and monitoring purposes, which would be crucial to limiting the spread of MRSA.

## Introduction

*Staphylococcus aureus* is a gram-positive commensal bacterium that commonly inhabits different parts of the body including nostrils and axilla [1]. MRSA is a multi-drug resistant strain of *S*. *aureus* that has gained global attention, particularly due to a large number of refugees and asylum seekers who come from regions with high MRSA prevalence [2]. The *mec*A gene, which is carried on a staphylococcal chromosomal cassette (*SCCmec*), produces penicillin-binding protein A (PBPa) that confers methicillin resistance [3, 4]. Infections caused by MRSA can range from superficial skin and soft tissue infections to life-threatening infections such as bacteremia, endocarditis, necrotizing pneumonia, necrotizing fasciitis, and osteomyelitis [5–8]. MRSA toxins also play a role during infections and conditions such as toxic shock syndrome [4, 6] staphylococcal scarlet fever, staphylococcal scalded-skin syndrome, and food poisoning [6]. Different virulence factors that are encoded by different genes play a major role during pathogenesis. Such factors include panton-valentine leucocidin toxins (PVL, encoded by *lukS/F-PV* and *lukE/D* genes) [5, 7, 8], exfoliative toxins (*etb* and *eta*), arginine catabolic mobile element (ACME, *arcA*), beta-hemolysin (*hlb*), toxic shock syndrome toxin-1 (TSST-1, *tst*) and accessory gene regulator (*agr*) [6, 7] and alpha-hemolysin (*hla*) [6].

Conflict escalation in Syria has led to many Syrian civilians fleeing for neighboring countries including the Kurdistan region of Iraq and subsequently to European countries. The majority of these refugees (approximately 109,352) are located in Duhok city, which is considered to be the main entry point due to its geographical location next to the Syrian and Turkish borders [9]. Overcrowded conditions, particularly in camps, may play a role in spreading infections caused by pathogens such as MRSA. There is limited data available about differences in MRSA virulence within the native Iraqi and Syrian refugee communities. As such, this study aimed to characterize the virulence profiles of MRSA found in the two communities by genotyping genes that encode virulence factors and toxins.

## Materials and methods

### Study samples

This study was conducted from January 2018 to October 2019. Frozen MRSA strains (125) that were isolated from students were used. The strains were obtained from healthy participants’ nares in both groups by using sterile swabs. Forty-nine subjects were Syrian refugees and 76 were from native Iraqi community of Duhok city, Iraq.

### Identity verification for methicillin-resistant *S*. *aureus*

#### Conventional detection of MRSA

The frozen isolates were recovered by culturing on a selective culture media, mannitol salt agar (Neogen company, UK), and produce bacterial colonies. The isolates were confirmed as *S*. *aureus* through tests for mannitol fermentation, gram stains and biochemical tests, and catalase and tube coagulase activity [10]. All isolates that were used were further tested for antibiotic susceptibility against oxacillin, and were confirmed to be MRSA. An agar dilution assay was performed on Mueller-Hinton agar (MHA) (Neogen, UK) containing oxacillin (6 μg/mL) and 4% NaCl. A bacterial suspension equivalent to a 0.5 McFarland standard was cultured on MHA plates and incubated under aerobic conditions overnight at 35 ˚C. The results were interpreted according to the Clinical and Laboratory Standards Institute (CLSI) guidelines [11].

#### DNA extraction

PureLink Genomic DNA Mini Kit (Invitrogen by Thermo Fisher Scientific, USA) was used for extraction and purification of genomic DNA from MRSA isolates. The extraction and purification was performed according to manufacturer’s instructions.

### Polymerase chain reaction (PCR) of MRSA markers

#### PCR amplification of Staphylococcus genus-specific 16s rRNA gene

For all isolates that had been confirmed to be MRSA by conventional methods, singleplex PCR was performed to detect a 16s rRNA gene that is specific for the *Staphylococcus* genus. Total PCR volume was 30 μl and included DNA template (1μl), hot start prime *Taq* DNA Polymerase (1 units/10μl), 2x reaction buffer, MgCl_2_ (4 mM), enzyme stabilizer, loading dye, and dATP, dCTP, dGTP, dTTP (pH 9.0, 0.5 mM each), and primers Staph756F and Staph750R (both 10 pM, sequence in Table 1) [12]. For PCR, the conditions were initial heat denaturation at 94°C for 2 min, followed by 35 cycles of denaturation at 94°C for 60 s, annealing at 55°C for 30 s, extension at 72°C for 90 s, and final extension at 70°C for 4 min. ATCC 29213 was used as a positive control for the 16s rRNA gene.

**Table 1:** Primer sequences and PCR product size.

Genes	Primer sequence(s)	Size (bp)	References
**16s rRNA**	Staph756F (5’-AACTCTGTTATTAGGGAAGAACA-3’)	756	McClure *et al*., [12]
	Staph750R (5’-CCACCTTCCTCCGGTTTGTCACC-3’)		
***mec*A**	MecA1 (5′ -GTAGAAATGACTGAACGTCCGATAA-3′)	310	
	MecA2 (5′-CCAATTCCACATTGTTTCGGTCTAA-3′)		
***lukS/F-PV***	Luk-PV-1 (5′-ATCATTAGGTAAAATGTCTGGACATGATCCA-3′)	433	
	Luk-PV-2 (5′-GCATCAAGTGTATTGGATAGCAAAAGC-3′)		
***arc*A**	arcA-F (5’-GCAGCAGAATCTATTACTGAGCC-3’)	513	Zhang *et al*. [13]
	arcA-R (5’-TGCTAACTTTTCTATTGCTTGAGC-3’)		
***lukE-lukD***	LUKDE-1 (5’-TGAAAAAGGTTCAAAGTTGATACGAG-3’)	269	Jarraud et al. [6]
	LUKDE-2(5’-TGTATTCGATAGCAAAAGCAGTGCA-3’)		
***hla***	HLA-1 (5′- CTGATTACTATCCAAGAAATTCGATTG-3´)	209	
	HLA-2 (5′- CTTTCCAGCCTACTTTTTTATCAGT-3´)		
***hlb***	HLB-1 (5′ -GTGCACTTACTGACAATAGTGC-3´)	309	
	HLB-2-2 (5′ -GTTGATGAGTAGCTACCTTCAGT		
***tst***	GTSSTR-1 (5′- ACCCCTGTTCCCTTATCATC-3′)	326	Mehrotra et al. [3]
	GTSSTR-2 (5′- TTTTCAGTATTTGTAACGCC-3′)		
***eta***	GETAR-1 (5′- GCAGGTGTTGATTTAGCATT-3′)	93	
	GETAR-2 (5′- AGATGTCCCTATTTTTGCTG-3′)		
***etb***	GETBR-1 (5′- ACAAGCAAAAGAATACAGCG-3′)	226	
	GETBR-2 (5′- GTTTTTGGCTGCTTCTCTTG-3′)		
***agr***	Pan F(5′-ATGCACATGGTGCACATGC-3′)		Gilot et al.[14]
*agr* group I	agr1 R (5′-GTCACAAGTACTATAAGCTGCGAT-3′)	441	
***agr* group II**	agr2 R (5′-TATTACTAATTGAAAAGTGGCCATAGC-3′)	575	
*agr* group III	agr3 R (5′-GTAATGTAATAGCTTGTATAATAATACCCAG3′)	323	
***agr* group IV**	agr4 R(5′-CGATAATGCCGTAATACCCG-3′)	659	

#### PCR amplification of mecA gene

The *mec*A gene was amplified using *mec*A1 and *mec*A2 primers, with the sequences provided in Table 1 [7]. PCR was carried out as previously described for the 16s rRNA gene. PCR conditions that were used were initial heat denaturation at 94°C for 5 min, followed by 30 cycles of denaturation at 94°C for 45 s, annealing at 60°C for 45 s, extension at 72°C for 90 s, and final extension at 72°C for 6 min. ATTC 43300 was used as a positive control for the *mec*A gene.

### Genotyping of virulence genes by PCR

#### Panton-valentine leukocidin (PVL) gene

All MRSA isolates were tested for the PVL genes *Luk-PV-1* and *Luk-PV-2* (primer sequences in Table 1) [12]. The PCR was made up as previously described for the 16s rRNA gene. PCR conditions (touch down PCR) used were as follows: initial heat denaturation at 94°C for 10 min, 10 cycles of denaturation at 94°C for 45 s, annealing at 55°C for 45 s, and extension at 72°C for 75 s. This was followed by another 20 cycles of denaturation at 94°C for 45 s, annealing at 50°C for 45 s, and extension at 72°C for 75 sec, with a final extension at 72°C for 6 min.

#### Arginine Catabolic Mobile Element (ACME) *arc*A gene

PCR was used to amplify the *arc*A gene present on ACME (sequences for primers *arc*A-F and *arc*A-R in Table 1) [13]. The PCR was made up as previously described. PCR conditions (touch down PCR) used were as follows: initial heat denaturation at 94°C for 4 min, 10 cycles of denaturation at 94°C for 30 s, annealing at 60°C for 30 s, and extension at 72°C for 45 s. This was followed by another 25 cycles of denaturation at 94°C for 30 s, annealing at 52°C for 30 s, and extension at 72°C for 45 s, with a final extension at 72°C for 6 min.

#### LukE-LukD leucocidin (*lukE-lukD*) gene

PCR was used to amplify the *lukE-lukD* gene (sequences for primers LUKDE-1 and LUKDE-2 in Table 1) [6]. The PCR was made up as previously described. PCR conditions used were as follows: initial heat denaturation at 94°C for 30 s, followed by 30 cycles of denaturation at 94°C for 30 s, annealing at 55°C for 30 s, extension at 72°C for 30 s and a final extension at 72°C for 2 min.

#### Alpha-hemolysin (*hla*) gene

The gene encoding alpha-hemolysin toxin, *hla*, was amplified using set of primes (HLA-1 and HLA-2 that are in Table 1) [6]. The PCR was made up as previously described for the 16s rRNA gene. The thermocycling conditions were included: initial denaturation at 94°C for 4 min, followed by 30 cycles of denaturation at 94°C for 30 s, annealing at 55°C for 30 s, extension at 72°C for 30 s, and a final extension at 72°C for 5 min.

#### Beta-hemolysin (*hlb*) gene

The gene encoding Beta-hemolysin, *hlb*, was amplified using singleplex PCR (sequences for primers HLB-1 and HLB-2-2 are in Table 1) [6]. The PCR was made up as previously described. PCR conditions were as follows: initial denaturation at 94°C for 30 s, followed by 30 cycles of denaturation at 94°C for 30 s, annealing at 55°C for 30 s, extension at 72°C for 30 s and a final extension at 72°C for 2 min.

#### Toxic shock syndrome toxin-1 (*tst*) gene

PCR was used to amplify the *tst* gene that encodes toxic shock syndrome toxin. Total PCR volume was 30 μl and included Crystal Hot Start Master mix (Jena Bioscience, Germany) composed of 2 ×, Hot Start *Taq* polymerase, nucleotides (dATP, dCTP, dGTP, dTTP), KCl, (NH_4_)_2_SO_4_, MgCl_2,_ density reagent, enhancing and stabilizing additives, DNA template (1 μl) and forward and reverse primers (10 pM, primer sequences in Table 1) [7]. PCR conditions were as follows: initial heat denaturation at 94°C for 5 min, followed by 35 cycles of denaturation at 94°C for 2 min, annealing at 57°C for 2 min, extension at 72°C for 1 min, and a final extension at 72°C for 7 min.

#### Typing of accessory gene regulator (*agr*) genes

Multiplex PCR was used to amplify all types (I-IV) of *agr* genes that encode accessory gene regulator elements (primers used listed in Table 1) [14]. Total PCR volume was 40μl and included the same components as those previously described for PCR of the *tst* gene. PCR conditions were as follows: initial heat denaturation at 95°C for 5 min, followed by 35 cycles of denaturation at 94°C for 30 s, annealing at 57°C for 90 s, extension at 72°C for 60 s, and a final extension at 68°C for 10 min.

#### Exfoliative toxin A (*eta*) and Exfoliative toxin B (*etb*) genes

Multiplex PCR was used to amplify *eta* and *etb* genes (all primers listed in Table 1) [3]. Total PCR volume was 40μl and included the same components as those previously described for PCR of the *tst* gene. PCR conditions were as follows: initial heat denaturation at 94°C for 5 min, followed by 35 cycles of denaturation at 94°C for 2 min, annealing at 57°C for 2 min, extension at 72°C for 1 min, and a final extension at 72°C for 7 min.

### Preparation of agarose gel for visualization of PCR products by electrophoresis

PCR products for all genes were visualized by 2% agarose gel (GeNet Bio, Korea) electrophoresis. Agarose powder (2%) was heated and homogenously dissolved in TAE buffer. Safe Dye with green fluorescence (GeNet Bio, Korea) was added to the gel. Amplified PCR products were then separated on the gel by electrophoresis (80 V, 45 min) and compared to a DNA marker ladder (GeNet Bio, Korea). The gel was exposed to UV to visualize the bands (expected amplicon size sin Table 1).

### Statistical analysis

Minitab software version 17 (Minitab, LLC) was used for data analysis. Data were presented as frequencies and percentages. The chi-square test was used for comparison between groups and the level of significance was set at *P*-value < 0.05.

### Ethics considerations

Ethics approval to collect data from subjects was obtained from the university scientific committee of the College of Medicine, University of Duhok, the Board of Relief and Humanities Affairs (BRHA) for refugee’s affairs in Duhok, and the United Nations High Commissioner for Refugees (UNHCR). Prior to data collection, signed consent was obtained from all participants. This study was performed in accordance with the ethical standards as laid down in the 1964 Declaration of Helsinki and its later amendments or comparable ethical standards.

## Results

The isolates were tested for oxacillin susceptibility and their identity was confirmed to be MRSA by PCR for *mec*A and Staphylococcal genus- specific 16s rRNA genes. We used PCR to amplify various genes that encoded Staphylococcal virulence factors of MRSA, including *pvl*, *arc*A, *tst*, *lukE/lukD*, *hlb*, *eta*, *etb* and *agr* genes. The prevalence rate (Table 2) for *arc*A in the native Iraqi community (56.58%) was significantly higher than (*p* = 0.008) that in the Syrian refugee community (32.66%). The prevalence rate of *lukE-lukD* gene was also significantly higher (*p* = 0.001) in the Iraqi community (82.89%) compared to the Syrian refugee community (57.14%). Further, prevalence of *hla* MRSA in the Iraqi community was (93.4%) and in the Syrian refugee community was (71.4%); (*p* = 0.0008). However, no difference was found between both study groups for *pvl*, *tst*, *eta*, *etb* and *hlb* genes (Table 2). Additionally, we identified the common types of MRSA *agr* groups (Table 3) as *agr* types I (76.1% and 44.9% in the Iraqi and Syrian refugee communities respectively) and III (10.5% and 18.37% respectively).

**Table 2:** Prevalence of genes encoding virulence factors and toxins and comparison of MRSA in the native Iraqi and Syrian refugee communities.

Genes encoding virulence factors and toxins	Positive (+ve)/ negative (-ve)	Iraqi community isolates *n* (%)	Refugee isolates *n* (%)	P-value
***pvl***	+ve	3 (3.95)	1 (2.04)	.554
	-ve	73 (96.05)	48 (97.96)	
***arcA***	+ve	43 (56.58)	16 (32.66)	.008
	-ve	33 (43.42)	33 (67.34)	
***tst***	+ve	61 (80.26)	44 (89.8)	.155
	-ve	15 (19.74)	5 (10.2)	
***lukE-lukD***	+ve	63 (82.89)	28 (57.14)	.001
	-ve	13 (17.11)	21 (42.86)	
***hla***	+ve	71 (93.4)	35 (71.4)	.0008
	-ve	5 (6.6)	14 (28.6)	
***hlb***	+ve	73 (96.1)	48 (97.95)	.554
	-ve	3 (3.95)	1 (2.04)	
***eta***	+ve	0 (0)	0 (0)	0.99
	-ve	76 (100)	49 (100)	
***etb***	+ve	1 (1.31)	0 (0)	0.42
	-ve	75 (98.68)	49 (100)	

*n;* number

**Table 3:** Genotyping of *agr* group among native Iraqi and Syrian refugees communities in Duhok city, Iraqg.

*agr* group	Iraqi community MRSA isolates *n*(%)	Syrian refugee community MRSA isolates *n(%*)	P-value
***agr* I**	51 (76.1)	22 (44.9)	0.20
***agr* II**	4 (5.26)	1 (2.04)	0.65
***agr* III**	8 (10.52)	9 (18.37)	0.28
***agr* IV**	0 (0)	0 (0)	0.99
**Double-positives**			
***agr* I + *agr* II**	0	2 (4.08)	0.16
***agr* I + *agr* III**	0	1 (2.04)	0.39
**Un-typable**	13 (17.1)	14 (28.57)	0.23
**Total**	76	49	

*n;* number

## Discussion

MRSA is a major health threat in developing countries due to its ability to cause life-threatening infections. In this study differences in various virulence factors were characterized in Iraqi and Syrian refugee communities. PVL toxin is responsible for severe infections caused by community acquired MRSA (CA-MRSA) [15]. PVL is encoded by *lukS-PV* and *lukF-PV* genes and acts as a toxin by creating pores in leukocytes’ cell membrane, leading to cell death [5, 16]. We found that the prevalence of PVL-positive MRSA was (3.95%) among native Iraq, and was higher than that in Syrian refugees (2.04%). The rates we found in both communities were lower than what was previously reported in Iraq (19% and 10%) [17, 18], Turkey (6.9%) [19], and China (47.8%) [20]. Prevalence rates of PVL-positive MRSA among Syrian refugees was comparable with Palestine (2.5%) [21] and Brazil (2.4%) [22]. With the limited data available about MRSA- PVL prevalence in Syria, we believe that this study can act as a basis for comparison in further studies conducted within the region.

*Staphylococcus aureus* can adapt to environments and hosts efficiently by the help of various virulence factors and resistance genes that play the major role in pathogenicity. The genetic information of these virulence factors and resistant determinants are exchanged between different bacterial species by a mean of mobile genetic elements (MGEs). MGEs may include insertion sequences, transposons, bacteriophages, plasmids, pathogenicity islands, and chromosome cassette [23].

ACME is a mobile genetic element that is novel to USA300 MRSA strains and encodes the *arc*A gene [24]. We found a significantly higher rate of *arc*A-positive MRSA in Iraqi samples compared to Syrian refugee samples. A previous study observed, 10% of isolates from healthy subjects and 3.8% of CA-MRSA isolates from clinical samples were positive for *arc*A in Iraq [25]. This could potentially indicate a 5-fold increase in prevalence rate of *arc*A-positive MRSA in Iraq. This would be a cause for alarm, and further studies are urgently required to determine if prevalence rate is rapidly increasing. We also found that the prevalence rate of *arc*A-positive MRSA in both communities was higher than that in Palestine (18.8%) [4], the USA (18%) [7] and Armenia (15%) [26]. To the best of our knowledge, this is the first reported ACME prevalence rate in a Syrian population, and further studies with larger sample sizes, and community and hospital isolates need to be conducted.

The superantigen TSST-1 is an extracellular protein that causes toxic shock syndrome and is encoded by the *tst* gene [27]. *Tst* gene is encoded by a highly mobile pathogenicity island (SaPI) [28]. Our results showed *tst* prevalence rates of 80.26% and 89.8% in MRSA isolates from the Iraqi and Syrian refugee communities respectively. These rates were higher than what has been reported in Egypt (58.5%) [29], Palestine (23.2%) [4], the USA (2%) [7], China (8.7%) [20], and Iran [30]. The high prevalence rate of *tst* gene in both groups might be due to recent transfer of SaPI between strains. Besides, there is a lack of data regarding *tst*-positive MRSA in this region, and further studies are required to determine the precise reason for the exceptionally high prevalence rates observed.

Alpha-hemolysin toxin is encoded by *hla* gene. It has a cytotoxic effect on host cells (erythrocytes) by forming pore in the cell membrane. It plays a major role in *S*. *aureus* in causing various clinical conditions such as pneumonia, septicemia, septic arthritis, brain abscess and eye infections [31]. We found that the prevalence of *hla* gene in MRSA isolates was significantly higher in the Iraqi community (93.4%) compared to Syrian refugees (71.4%). Our results for Iraqi community were close to a previous study conducted in Iraq where they found (95%) of strains positive to the gene [32] and similar to Iran where (93.15%) of isolates were positive for *hla* gene [33]. No data was found about the prevalence of this gene in Syria.

Beta-hemolysin, is associated with respiratory and eye infections, and is encoded by the *hlb* gene, producing the enzyme that destroys erythrocytes and releases free hemoglobin [31]. Our results showed that prevalence rates for *hlb*-positive MRSA were (96.1%) and (97.95%) in the Iraqi and Syrian refugee communities respectively. These rates were higher than that found in China 90.3% [20], and was comparable to the prevalence rate in the USA (96%) [7].

For potential to produce leucocidin toxin, we measured prevalence of *lukE- lukD* in MRSA isolates and found that rate of prevalence was significantly higher in the Iraqi community (82.89%) compared to Syrian refugees (57.14%). Such a high rate of positivity in Iraq might indicate that it is endemic, however, further studies are needed to verify this. No data are available for this gene in Syria and continuous monitoring is mandated in this country.

Exfoliative toxins, encoded by *eta* and *etb* genes, are responsible for skin and cutaneous tissue infections and scalded skin syndrome [3, 4]. In this study, neither *eta* nor *etb* were found in MRSA isolates from Syrian refugees. This is similar to MRSA strains in China [20] and the USA [7]. While 1.31% of MRSA isolates from the Iraqi community carried the *etb* gene, *eta* was not detected. Such a discrepancy in results might be attributable to differences in recruited participants and/or geographic location.

Most virulence-associated genes in *S*. *aureus* are expressed as extracellular proteins. Their expression are controlled by the *agr* genes, which are divided into four groups [6, 34]. We characterized *agr* types of MRSA isolates from the Iraqi and Syrian communities by multiplex PCR, and found that the majority were *agr* type I, *agr* type III and *agr* type II in both Iraqi (76.1%, 10.52%, and 5.26% respectively) and Syrian refugee (44.9%, 18.37%, and 2.04% respectively) communities. No type IV *agr* was detected in both groups. In contrast, another study found that *agr* type II (58.5%) and *agr* type I (25.7%) were the dominant groups found in Egypt [29]. A different study also found that carried out among the majority of MRSA from neonates in the USA were also *agr* type II (67%) and *agr* type I (30%) [7]. Further studies are needed to identify the un-typable *agr* strains by sequencing.

Although our PCR-based study is valuable and can be utilized as a baseline report, it has limitations. PCR can only identify the presence or absence of the genes regardless their functionality. Gene expression analyses is required to study the formation of a gene product from its coding gene. Additionally, strains used in this study were isolated from healthy volunteers rather than clinical samples. To investigate the role of such genes in the pathogenesis of infection, strains should be isolated from clinical samples and the correlation between clinical outcomes and gene expression can be studied.

## Conclusion

MRSA is a serious health threat and has various virulence factors associated with different strains. Through genotypic study, we found remarkably high rates of virulence genes in both communities. We also found that prevalence rates of *arcA*, *lukE-lukD* and *hla* were significantly higher in the Iraqi community compared to the Syrian refugee community. Additionally, the most common *agr* types in both communities were *agr* type I and III. Our results could be used as a basis of comparison for further studies. Further study of the genetic relatedness of MRSA strains found in the region should be conducted for epidemiological purposes and continuous vigilance is imperative to control the spread of MRSA.
